# Biophysical Studies of Amyloid-Binding Fluorophores to Tau AD Core Fibrils Formed without Cofactors

**DOI:** 10.3390/ijms25189946

**Published:** 2024-09-15

**Authors:** Daniela P. Freitas, Joana Saavedra, Isabel Cardoso, Cláudio M. Gomes

**Affiliations:** 1 BioISI—Instituto de Biosistemas e Ciências Integrativas, Faculdade de Ciências, Universidade de Lisboa, 1749-016 Lisboa, Portugal; dpfreitas@ciencias.ulisboa.pt; 2 Departamento de Química e Bioquímica, Faculdade de Ciências, Universidade de Lisboa, 1749-016 Lisboa, Portugal; 3 i3S—Instituto de Investigação e Inovação em Saúde, Universidade do Porto, 4200-135 Porto, Portugal; joana.saavedra@i3s.up.pt (J.S.); icardoso@ibmc.up.pt (I.C.); 4 IBMC—Instituto de Biologia Molecular e Celular, Universidade do Porto, 4200-135 Porto, Portugal; 5 ICBAS—Instituto de Ciências Biomédicas Abel Salazar, Universidade do Porto, 4050-313 Porto, Portugal

**Keywords:** tau aggregation, microtubule-binding repeats, amyloid-binding fluorophores, fibril aggregation kinetics

## Abstract

Tau is an intrinsically disordered protein involved in several neurodegenerative diseases where a common hallmark is the appearance of tau aggregates in the brain. One common approach to elucidate the mechanisms behind the aggregation of tau has been to recapitulate in vitro the self-assembly process in a fast and reproducible manner. While the seeding of tau aggregation is prompted by negatively charged cofactors, the obtained fibrils are morphologically distinct from those found in vivo. The Tau AD core fragment (TADC, tau 306–378) has emerged as a new model and potential solution for the cofactor-free in vitro aggregation of tau. Here, we use TADC to further study this process combining multiple amyloid-detecting fluorophores and fibril bioimaging. We confirmed by transmission electron microscopy that this fragment forms fibrils after quiescent incubation at 37 °C. We then employed a panel of eight amyloid-binding fluorophores to query the formed species by acquiring their emission spectra. The results obtained showed that nearly all dyes detect TADC self-assembled species. However, the successful monitoring of TADC aggregation kinetics was limited to three fluorophores (X-34, Bis-ANS, and pFTAA) which yielded sigmoidal curves but different aggregation half-times, hinting to different species being detected. Altogether, this study highlights the potential of using multiple extrinsic fluorescent probes, alone or in combination, as tools to further clarify mechanisms behind the aggregation of amyloidogenic proteins.

## 1. Introduction

Tau is an intrinsically disordered protein that is involved in a myriad of neurodegenerative pathologies, all grouped under the umbrella term “tauopathies” that includes Alzheimer’s Disease (AD). Globally, more than 55 million people suffer from dementia, with estimates of 7 million new cases per year, resulting in a massive societal burden [1]. Despite the differences between these diseases, they all share as a hallmark the progressive accumulation of tau aggregates in the brain [2]. Even though much research has been pursued to understand the aggregation mechanisms of tau, one major limitation is the difficulty in generating tau aggregates in vitro spontaneously, in a reproducible and quick manner. This seems to be a consequence of the structure of tau: while having a high aggregation propensity, tau has a mostly positive aggregation-prone region (the Microtubule-Binding Repeats region, MTBR, tau_244–372_) flanked by the N-terminal and C-terminal segments in a paperclip-like shape [3], which complicates its self-assembly unless it is in the presence of a negatively charged cofactor such as heparin or RNA [4,5]. Even though the use of these molecules accelerates the aggregation process [6,7,8], the end species tend to significantly differ in structure from those found in patient brains [9,10]. For this reason, one main goal in the field has been to develop in vitro models that are able to more closely recapitulate tau aggregation events like those found in vivo, without the need of accelerating cofactors.

A common tool to explore the in vitro aggregation of tau and of other amyloidogenic proteins has been the use of small fluorescent probes that bind species formed during the self-assembly process, changing their fluorescence properties upon binding. This approach is capable of providing useful information on the aggregation process, based on differences in emission intensity, fluorescence lifetime, or changes in emission wavelength [11]; it is thus possible to monitor the in vitro aggregation kinetics but also to discriminate between different amyloid species by employing combinations of these probes [12,13]. As reviewed extensively by Aliyan et al. [11], most of these probes seem to interact with the typical cross-β structure present in mature amyloid fibers, though some have been designed to identify smaller oligomeric forms with varying selectivity and sensitivity. Consequently, they could serve as a powerful, fast, and inexpensive tool to gain understanding on amyloid formation.

**Figure 1:**
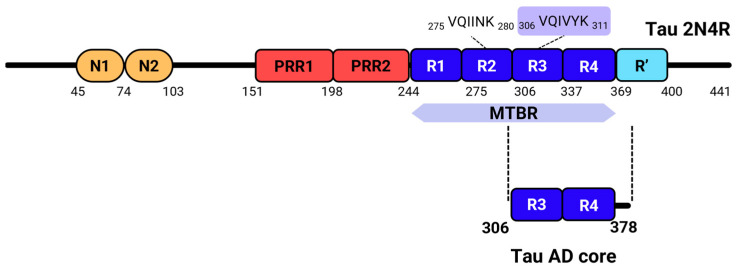
Tau structure. Full-length 2N4R tau contains two N-terminal domains (in orange), followed by two proline-rich regions (in red), and four microtubule-binding repeats (R1, R2, R3, and R4), which comprise nearly the entire microtubule-binding domain of tau. Within these repeats, the PHF6*/PHF6 hexapeptides, highlighted above the R2 and R3 repeats, play a crucial role in tau aggregation [18,19]. A final pseudo-repeat, R’, also contributes to tau’s interaction with microtubules and is located in the C-terminal region [20]. The Tau AD core, which forms the structured core of tau fibrils in Alzheimer’s Disease [15], primarily involves the R3 and R4 repeats and includes the PHF6 peptide.

In 2021, Rodriguez-Camargo and colleagues [14] reported the heparin-free aggregation of a tau fragment corresponding almost exactly to the core of the tau fibrils found in Alzheimer’s Disease (AD) [15]. In the same year, other groups also reported the cofactor-free aggregation of either full-length tau [16] or the tau AD core [17]. Based on this, we sought to replicate the cofactor-free aggregation of the tau AD core (TADC, tau_306–378_), which approximately corresponds to the R3 and R4 repeats of MTBR (Figure 1). We further aimed to characterize the resulting species using biophysical approaches, focusing on a fluorescence-based analysis to characterize the aggregates formed under such conditions. Here, we confirm the formation of fibrillar TADC species under cofactor-free conditions and provide spectral signatures of such conformers using a panel of fluorophores able to detect amyloids and oligomers: ThT, ThS, pFTAA, hFTAA, ANS, Bis-ANS, X-34, and Congo Red.

## 2. Results

### 2.1. TEM Analysis of Cofactor-Free Tau AD Core (TADC, Tau_306–378_) Aggregates

**Figure 2:**
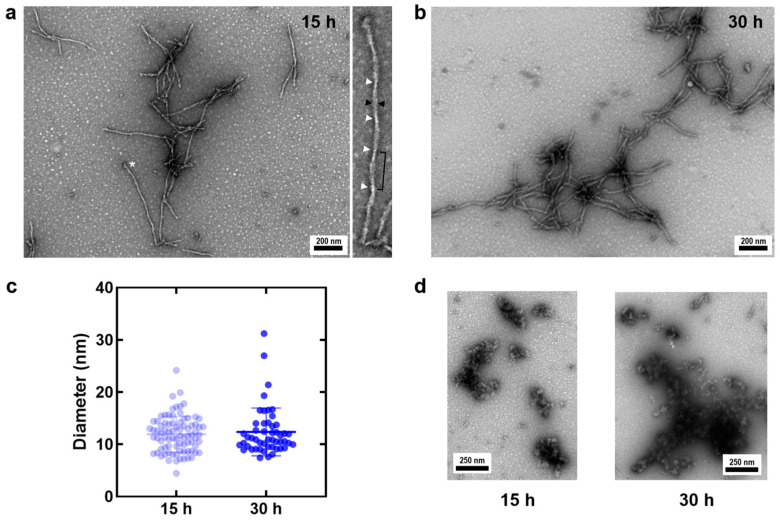
Negative-stain TEM images of TADC heparin-free aggregates. Fibrillar species observed at 15 h (**a**) and 30 h (**b**) during the cofactor-free, quiescent aggregation of 100 µM TADC monomers. In panel (**a**), the inset provides a zoomed view of the fibril marked with a white asterisk, showing how the measurements for apparent diameter (black arrowheads) were taken. White arrowheads point to potential fibril torsion regions, though these cannot be precisely identified in this study. (**c**) Measurement of apparent fibril diameters at 15 h (11.9 ± 3.4 nm, n = 82) and 30 h (12.4 ± 4.6 nm, n = 50). All measurements are reported as mean ± standard deviation. Globular, amorphous species were observed throughout the reaction, as shown in (**d**) for both 15 h and 30 h samples.

In order to obtain a biophysical and morphological characterization of TADC aggregates formed in the absence of aggregation-inducing cofactors, 100 μM TADC was incubated under quiescent conditions at 37 °C for up to 30 h. To evaluate the progression of the formed TADC aggregates along the reaction, we employed negatively stained TEM to visualize the morphology of the species formed after incubation during 15 h and 30 h, which roughly correspond to the mid-growth phase and to the plateau stage [14]. The analysis of the obtained images shows the formation of short fibrillar species irrespective of the incubation time analyzed (Figure 2a–c), in agreement with the fact that during amyloid fibril formation, fibrils are present at all times [21]. At the two examined time points, we could not detect a noticeable difference in the apparent diameter of these fibrillar structures (Figure 2c; 11.9 ± 3.4 nm at 15 h and 12.4 ± 4.6 nm at 30 h).

Some of the TADC fibrils exhibit a turn periodicity and apparent diameter reminiscent of paired helical filaments, which are reported to have an apparent diameter of 10–20 nm [22,23]. At 15 h, we observe fibrils with and without apparent periodical twists, the latter displaying uniform staining along the fibril axis, potentially indicating structures similar to straight filaments [23]. The fibrils tended to form clusters with some lateral packing, although no specific clustering pattern was evident.

Additionally, globular structures, possibly corresponding to tau oligomeric species, were observed at both 15 and 30 h (Figure 2d). These globular structures cluster into amorphous assemblies that are heterogeneous in size and shape; due to their nature, these conformers, as opposed to fibrils, typically result in darker staining with uranyl acetate. It remains unclear whether these represent non-growth-competent oligomers or oligomers still in the process of aggregating into fibrils, a variability previously reported in heparin-induced tau aggregation [24].

### 2.2. Amyloid-Binding Fluorescent Probes Show Different Sensitivities to TADC Aggregates

Amyloid-binding probes are useful tools to monitor the in vitro aggregation of amyloidogenic proteins, but their selectivity and sensitivity can vary significantly. After confirming the formation of fibrillar species under cofactor-free conditions, and considering conflicting reports on which amyloid-binding dyes can monitor the aggregation of similar tau fragments without inducers, we sought to determine which fluorophores could effectively detect TADC fibers formed in the absence of aggregation inducers. To achieve this, we utilized the panel of commonly used fluorescent probes listed in Table 1. We obtained the emission spectra of eight different probes—ThT, ThS, pFTAA, hFTAA, ANS, Bis-ANS, X-34, and Congo Red—both alone and in the presence of cofactor-free TADC species collected after 30 h of incubation. To ensure consistent results, the same volume of each stock solution was added to all samples, minimizing differences due to volume variations.

**Figure 3:**
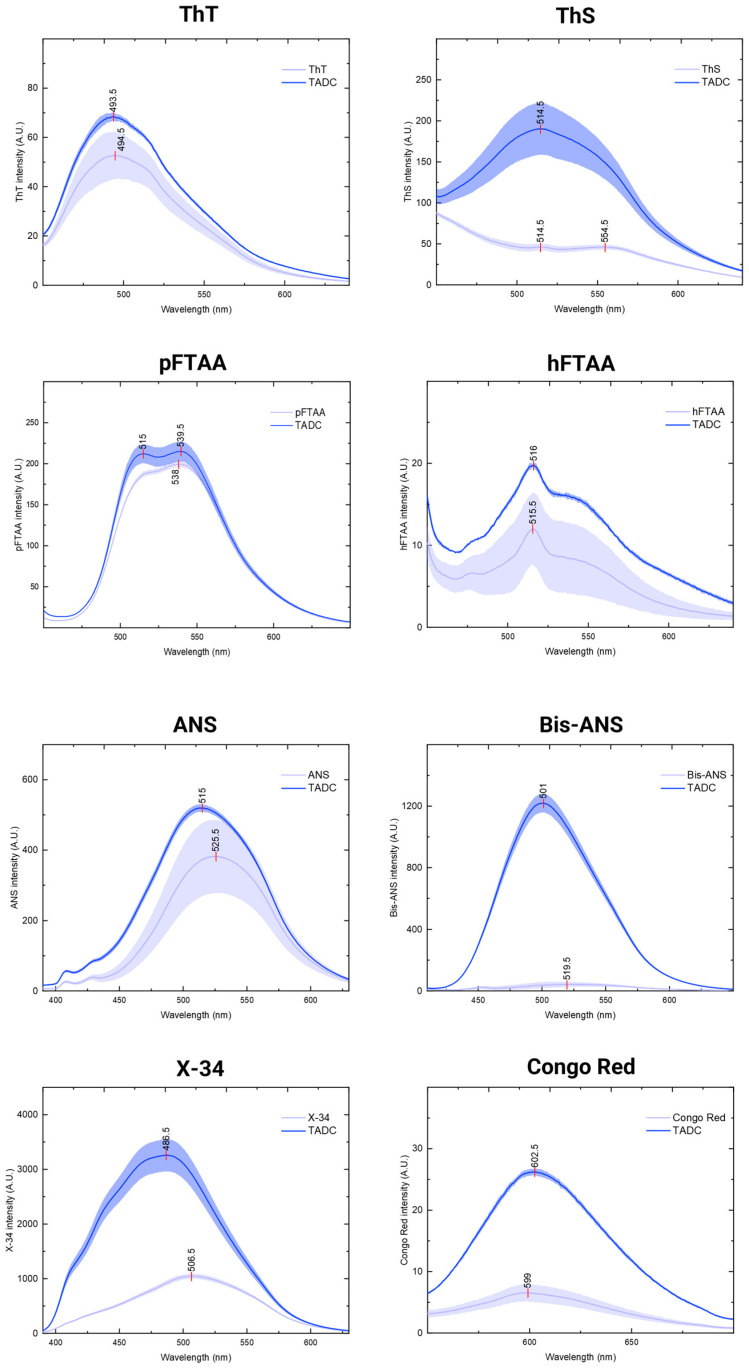
Emission spectra of fluorescent probes in the presence and absence of TADC aggregates. In total, 15 µM of TADC monomers was incubated for 30 h at 37 °C under quiescent conditions without inducers. Each dye was added to the endpoint samples, and spectra were obtained using the λ_ex_ specified in Table 1. Light blue spectra represent the emission of the probes alone, while dark blue spectra indicate the emission obtained by the probe in the presence of TADC aggregates. All spectra represent the average of triplicate measurements (line), with the standard deviation shown as a shaded area.

As shown in Figure 3, nearly all probes appeared to detect the formation of TADC aggregated species, in comparison to controls recorded with monomeric TADC (Figure S2). This was indicated by an increase in emission intensity when compared to the probe alone, by a shift in the spectral peak pattern, or by a combination of both effects. However, the sensitivity of these probes in detecting TADC species varies, with ThS, Bis-ANS, X-34, and Congo Red showing a particularly strong ability to detect TADC aggregates, as evidenced by their significant increase in emission intensity. Some of the probes, such as ANS, Bis-ANS, and X-34, show a blue shift in their emission upon interaction with TADC aggregates. Others, like ThS, pFTAA, and X-34, alter their spectral peak patterns, with new peaks emerging in the presence of TADC aggregates, a phenomenon previously observed with pFTAA with other amyloidogenic proteins [25].

Notably, the commonly used amyloid-binding dye ThT, along with hFTAA, exhibit only small changes in intensity and emission peaks, which could hint to a lower sensitivity towards TADC aggregates generated under cofactor-free conditions. This aligns with previous reports that mention difficulties in using ThT to monitor the aggregation of similar tau fragments without inducers [14,26]. It is worth noting that in reactions with heparin, all probes displayed distinct spectra compared to those obtained in its absence, with variations in both intensity and spectral shape (Figure S1). Notably, ThT exhibited a clear increase in intensity, indicating its potential for monitoring the self-assembly of TADC in the presence of heparin, whose TEM analysis evidences extensive fibril bundles (Figure S1, top panel). This suggests that the presence of heparin results in species that interact differently with the probes. In line with this, structural analysis revealed that heparin-induced tau filaments differ from those found in Alzheimer’s and Pick’s disease patients [9,10].

### 2.3. Cofactor-Free Aggregation of TADC Can Be Monitored by Selective Amyloid-Binding Probes

**Figure 4:**
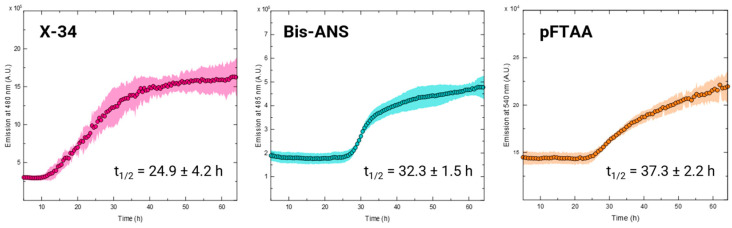
Kinetics of TADC cofactor-free aggregation. The aggregation kinetics of 15 µM of TADC monomers without inducers was monitored using X-34 (**left**), Bis-ANS (**middle**), and pFTAA (**right**), which yielded sigmoidal curves typical of fibril aggregation processes, albeit with different reaction half-times (t_1/2_). All curves correspond to the average of at least three replicates; ±standard deviation.

The continuous monitoring of the in vitro aggregation of amyloidogenic proteins offers valuable insights into their self-assembly mechanisms under various conditions and the impact of potential inhibitors, which is crucial for developing prospective treatments. Given the variability in emission observed previously and the potential for detection with different probes, we assessed the feasibility of monitoring TADC aggregation in the absence of an inducer. To investigate this, we incubated 15 µM of TADC monomers under the previously described conditions (37 °C, quiescent) with each fluorescent probe. Aggregation was effectively monitored using X-34, Bis-ANS, or pFTAA, as evidenced by the expected sigmoidal curves shown in Figure 4. It was, however, not possible to obtain reliable signal intensity with ThT, as reported before [11,14], but also not with ThS, hFTAA, ANS, and Congo Red, indicating that none of these probes is suitable to monitor cofactor-free TADC aggregation kinetics.

Among the three probes that yielded the prototypic sigmoidal-type amyloid aggregation kinetics, some differences were observed, including on the shape of the sigmoidal transitions and in the aggregation half-times (Figure 4). Although the latter are comparable, X-34 exhibited the shortest half-time (24.9 ± 4.2 h), followed by Bis-ANS (32.3 ± 1.5 h) and pFTAA (37.3 ± 2.2 h). It remains to be determined whether these variations reflect differences in dye sensitivity to the aggregated species or if they indicate varying selectivity of the dyes for specific conformers formed during cofactor-free TADC aggregation. For example, X-34 is known to be highly sensitive to amyloid-β oligomers [27], and further investigation is needed to assess if it shows similar sensitivity towards tau oligomers.

## 3. Discussion

Developing an in vitro cofactor-free aggregation model for tau that accurately reflects the properties of disease-derived aggregates has been a key goal in the field. One approach to achieve this has been the use of tau fragments involving MTBR, particularly those containing one or both of the PHF6*/PHF6 aggregation-prone motifs, to produce fibrillar species without the need for inducers. Among these, the fragment corresponding to the core of tau fibrils in Alzheimer’s Disease (TADC) has emerged as a promising model for inducer-free tau aggregation, reportedly capable of forming fibrils without the use of classical negatively charged cofactors such as heparin or RNA [14,17]. Here, we confirm that the TADC fragment can indeed form fibrils in vitro upon incubation at 37 °C under quiescent conditions (Figure 2). A comparison with fibrils generated with heparin suggests that the latter are apparently longer and tend to cluster into bundles (Figure S1).

Fluorescence spectroscopy is a widely used, versatile, and cost-effective technique that has significantly advanced our understanding of amyloid structures and behavior in vitro, ex vivo, and in vivo [11]. Noncovalent extrinsic fluorescent probes, in particular, are powerful tools for studying protein conformational changes and aggregate formation [28]. While thioflavin T (ThT) is commonly employed to investigate amyloid formation [29], our study confirmed that this probe is not very effective in detecting and monitoring the formation of TADC aggregates and fibrils. It has been proposed that the main mechanism by which ThT increases its emission intensity is a blockage of its ability to rotate upon binding to amyloid fibrils, influenced by the solvent’s polarity, viscosity, and temperature [30], though the presence of aromatic-rich motifs like Val-Phe or Tyr-Leu seem to play a role too [31]. We speculate that the lack of signal with ThT may be due to electrostatic repulsion between the predominantly positive TADC and the positively charged dye [32]. However, low ThT intensity has also been observed in other amyloidogenic proteins, such as the Aβ Japanese mutant ΔE22-Aβ1–39 [33], where it is believed to be due to structural characteristics of the fibrils that result in fewer apparent binding sites and lower overall affinity for the dye, a possibility that cannot be ruled out for TADC either.

Among all the dyes tested, Bis-ANS, pFTAA, and X-34 were particularly informative. Their spectra, after binding to TADC fibrillar species, showed variations in either intensity or shape, and they enabled the monitoring of aggregation kinetics. Bis-ANS, similar to its monomeric counterpart ANS, can interact with non-polar cavities in proteins [34]. However, it exhibits higher affinity than ANS [35] and appears capable of detecting both fibrils and oligomers [11,36]. This likely explains the significant increase in fluorescence emission observed upon interaction with TADC fibrils, as well as its potential to monitor aggregation. pFTAA did not exhibit a clear increase in emission intensity, but it did show a distinct peak increase at lower wavelengths, a phenomenon that has been previously reported for various fibrillar species [25]. X-34 is particularly interesting as it exhibits both a noticeable increase in intensity and alterations in its emission peak pattern, which also appears to differ, in more than just intensity, from the spectra obtained with heparin-induced fibrils. Previous reports have indicated that X-34 has a higher affinity for tau and Aβ fibrils than other dyes like ThT [37], which aligns with our findings and suggests that X-34 may be able to detect a broader array of amyloidogenic conformers. All three dyes proved suitable for monitoring TADC aggregation kinetics, each producing the characteristic sigmoidal trace with distinct lag phases and plateau stages, yet differing in their half-times. This variation suggests that the dyes may indeed detect different species, with some being more sensitive to early oligomers and protofibrils and others more apt to detect more mature fibrils.

In summary, the findings reported here underscore the potential to deepen our understanding of in vitro protein aggregation and amyloid fibril formation by utilizing a broader range of extrinsic fluorescent probes with potential applications in multiple protein systems [38]. This approach highlights the value of these tools, that can be used both individually or in combination [13,36], for providing detailed insights into the various species present throughout fibril formation.

## 4. Materials and Methods

### 4.1. Materials

All reagents were of the highest grade commercially available. For fluorescence-based assays, the following dyes were used: Thioflavin T (ThT, Sigma-Aldrich, St. Louis, MO, USA), Thioflavin S (ThS, Sigma-Aldrich, St. Louis, MO, USA), 8-Anilino-1-naphthalenesulfonic acid (ANS, Sigma-Aldrich, St. Louis, MO, USA), 4,4′-Dianilino-1,1′-binaphthyl-5,5′-disulfonic acid (Bis-ANS, Sigma-Aldrich, St. Louis, MO, USA), Congo Red (Sigma-Aldrich, St. Louis, MO, USA), X-34 (Sigma-Aldrich, St. Louis, MO, USA), and pentameric formyl thiophene acetic acid (pFTAA) and heptameric formyl thiophene acetic acid (hFTAA), kindly gifted by the VIB-Switch Laboratory (Prof. Joost Schymkowitz and Prof. Frederic Rousseau). When applicable, heparin (Sigma-Aldrich, St. Louis, MO, USA) was used, preparing a stock of 1.8 mg/mL, which was considered approximately equal to 100 µM for all pertinent calculations. A chelex resin (Bio-Rad, Hercules, CA, USA) was used to remove contaminant trace metals from all buffers.

### 4.2. Protein Expression and Purification

The plasmid encoding for TADC (tau 306–378) was obtained from Genscript (GenScript Biotech, Rijswijk, The Netherlands) by gene synthesis. For culture growth, pre-inoculums were prepared from glycerol stocks of transformed *E. coli* cells and incubated overnight in Luria–Bertani (LB) medium at 37 °C with agitation at 200 rpm. The medium was supplemented with 30 µg/mL chloramphenicol and 50 µg/mL kanamycin for recombinant selection. Subsequently, expression cultures were prepared by inoculating fresh LB medium with the pre-inoculum and allowing it to grow under the same conditions until an OD600 of approximately 0.6–0.8 was reached. Protein expression was then induced by adding 0.5 mM IPTG, followed by incubation for 4 h. Cells were harvested by centrifugation at 8000× *g* for 10 min at 4 °C using a JA-14 rotor, and the pellet was washed 1× with TBS buffer. For purification, the pellet was resuspended with lysis buffer (50 mM Tris-HCl pH 7.4, 50 mM NaCl, 1 mM MgCl_2_, and 1 mM DTT), adding 1 protein inhibitor cocktail tablet (Roche, Basel, Switzerland), 1 mM PMSF, and 4 mg/mL DNAse until a fluid homogenate was obtained. Cells were lysed using a sonicator set to 40% amplitude and 200 W, with cycles of 30 s ON and 30 s OFF for a total of 3 min. This was followed by three freeze–thaw cycles, alternating between liquid nitrogen and a water bath. The lysate was then centrifuged at 20,000 rpm for 1 h at 4 °C using a JA-25.50 rotor. The supernatant was recovered, heated at 80 °C for 5 min, cooled in ice for 10 min, and centrifuged again under the same conditions. The resulting supernatant was recovered and filtered. The fragment was purified from this sample by cation exchange chromatography, injecting the sample on a HiPrep SP FF 16/10 column (Cytiva, Marlborough, MA, USA) at 2 mL/min in Buffer A (50 mM Tris-HCl pH 7.4 + 50 mM NaCl). Once the unbound fraction was removed, the protein was eluted with a single-step shift to Buffer B (50 mM Tris pH 7.4 + 100 mM NaCl). Peaks were recovered and their purity checked by SDS-PAGE (8% gels). The corresponding TADC peak was concentrated using an Amicon centrifuge filter (Merck Millipore, Burlington, MA, USA) with a 3K cutoff in 50 mM Ammonium Bicarbonate buffer pH 7.4, lyophilizing it afterwards. TADC monomers were obtained by size-exclusion chromatography using a Superdex 75 Tricorn column (Cytiva, Marlborough, MA, USA), equilibrated with 50 mM Tris pH 7.4, after resuspending the lyophilized protein in 7.5 M urea with 50 mM DTT for 2–3 h, The monomer peak was aliquoted, flash-frozen, and stored for use.

### 4.3. Transmission Electron Microscopy

Samples for transmission electron microscopy (TEM) were prepared at 15 h and 30 h of TADC aggregation reactions without aggregation-inducing cofactors, by incubating 100 µM of TADC in 20 mM sodium phosphate buffer, pH 8, with 1 mM DTT and 1.1 mM CaCl_2_. Grids were prepared by letting 5 µL sample aliquots adsorb for 5 min to carbon-coated collodion film supported on 300-mesh copper grids (Electron Microscopy Sciences, Hatfield, Pennsylvania), and were negatively stained twice with 1% (m/v) uranyl acetate (Electron Microscopy Sciences, Hatfield, Pennsylvania). Grid washing before staining, to remove phosphate buffer to avoid dark precipitates, was attempted but fibrillar materials were lost. Grid visualization and image acquisition were performed with a JEOL 817 JEM-1400 transmission electron microscope operated at 80 kV equipped with an Orious Sc1000 digital camera. Fibril diameter was measured manually employing the ImageJ software (version 1.54f).

### 4.4. Fluorescence Measurements

Emission spectra for monomers and end-point species of TADC aggregation, with and without heparin, were obtained by preparing a master mix of 15 µM TADC in 20 mM sodium phosphate buffer pH 8.0, with 1 mM DTT and 1.1 mM CaCl_2_, with 0.94 µM heparin (6.25%) if needed. Part of this mix was taken to monitor aggregation by adding 2 µM X-34 under the conditions mentioned previously, as a control. The rest was placed in wells at 37 °C until the control reached the plateau (30 h for cofactor-free aggregations, and 3 h for aggregations with heparin). Once finished, the plates were placed in ice and spectrum measurements were taken for end-point species mixed with each of the following dyes in triplicate: 65 µM ThT, 20 µM ThS, 0.5 µM pFTAA, 0.5 µM hFTAA, 100 µM ANS, 3.5 µM BisANS, 50 µM Congo Red, or 2 µM X-34. Stocks for each dye were prepared so that the volume added to each sample was the same, to avoid variations in signal intensities due to differences in concentration. Emission spectra were taken by employing the following excitation wavelengths: 440 nm for ThT, ThS, pFTAA, and hFTAA; 375 nm for ANS; 395 nm for BisANS; 370 nm for X-34; and 497 nm for CR. The spectra were obtained with a Jasco FP-8200 Spectrofluorometer (Jasco, Japan) at 20 °C with medium sensitivity, bandwidths of 5 nm for both excitation and emission, and a scan speed of 200 nm/min, averaging 3 accumulations per spectrum.

### 4.5. Fluorescence-Monitored Aggregation Assays

Aggregation kinetics were analyzed by recording the fluorescence intensity as a function of time in a CLARIOstar Plus (BMGLabtech, Ortenberg, Germany) plate reader. Low-binding HTS 96-well microplates (Corning, Corning, NY, USA) and low-binding reaction-mix tubes (Axygen, Union City, CA, USA) were employed. TADC aggregation was monitored following previously published procedures for the cofactor-free aggregation of a similar fragment [14]. Namely, 15 µM TADC was monitored in 20 mM sodium phosphate buffer pH 8.0, with 1 mM DTT, 1.1 mM CaCl_2_, and 2 µM X-34, letting it aggregate at 37 °C in quiescent conditions. Multi-dye aggregation was followed by monitoring all dyes in parallel, using the Enhanced Dynamic Range function to establish the appropriate gain. The concentration of each dye and reading conditions used were as follows: 75 µM ThT, 30 µM ThS (both using λ_ex_: 440–15 nm, λ_em_: 480–20 nm), 0.5 µM of pFTAA, hFTAA (both using λ_ex_: 440–15 nm, λ_em_: 540–20 nm), 100 µM ANS, 2 µM X-34 (λ_ex_: 370–15 nm, λ_em_: 480–20 nm), 50 µM of Congo Red (λ_ex_: 497–15 nm, λ_em_: 614–20 nm), or 3.5 µM Bis-ANS (λ_ex_: 395–15 nm, λ_em_: 485–20 nm).

## Figures and Tables

**Table 1:** Panel of fluorophores employed in this study.

Fluorophore	Structure	λ_ex_ (nm)	λ_em_ Range (nm)
**ThT**	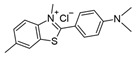	440	450–640
**ThS**	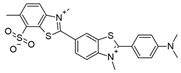	440	450–640
**pFTAA**	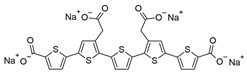	440	450–640
**hFTAA**	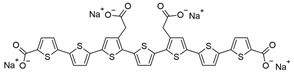	440	450–640
**ANS**	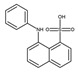	375	390–630
**Bis-ANS**	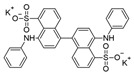	395	410–650
**X-34**	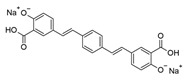	370	390–630
**Congo Red**	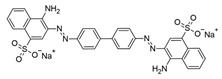	497	550–700

λ_ex_: excitation wavelength; λ_em_ range: emission wavelength range. Structures were drawn using ChemSketch, version 2022.1.2 (ACD/Labs, Toronto, ON, Canada).

## Data Availability

The original contributions presented in the study are included in the article/supplementary material, and further inquiries can be directed to the corresponding author.

## Supplementary Materials

The supporting information can be downloaded at: https://www.mdpi.com/article/10.3390/ijms25189946/s1.
