# Supplementary material for: Biophysical Studies of Amyloid-Binding Fluorophores to Tau AD Core Fibrils Formed without Cofactors
Source: Int J Mol Sci. 2024 Sep 15;25(18):9946. doi: 10.3390/ijms25189946 (PMC11432123; doi:10.3390/ijms25189946)
Supplement: Supplementary file 1 [file ijms-25-09946-s001.zip › ijms-3203793-supplementary.pdf]

Supplemental information

"Biophysical Studies of amyloid-binding fluorophores to Tau AD Core fibrils formed without cofactors"

Daniela Freitas, Joana Saavedra, Isabel Cardoso and Cláudio M. Gomes

International Journal of Molecular Sciences.

Figure S1

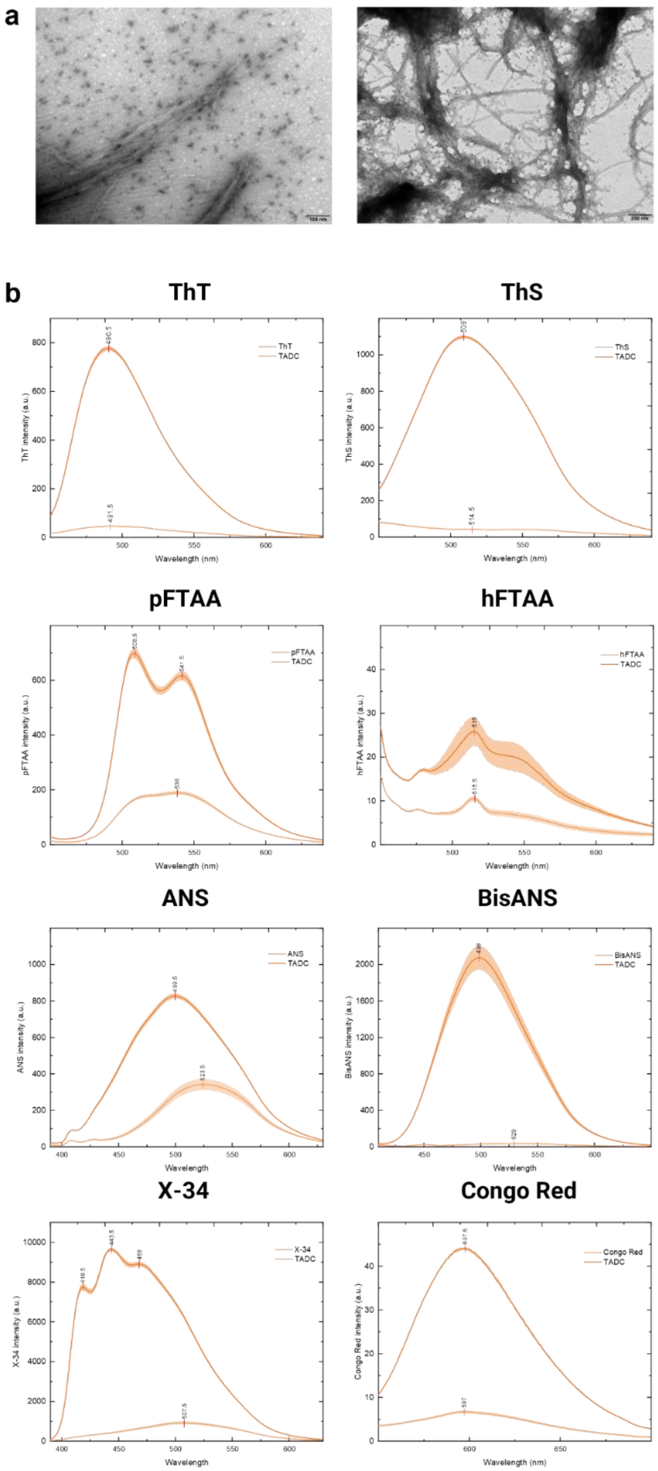

Figure S1. Biophysical characterization of TADC heparin-induced aggregates. a) TEM images of TADC endpoint aggregates after incubation with heparin. 100 µM of TADC monomers were incubated under quiescent conditions at 37 °C with 6.25 % heparin. Long fibrils are present, some of them showing twists without a clear crossover pattern, and in some cases appearing in bundles. b) Spectra of TADC heparin-induced aggregates with different amyloid-binding fluorescent probes. 15 µM of TADC monomers were incubated under quiescent conditions with 6.25 % of heparin for 3h at 37 °C. Each dye was added to the endpoint samples, and spectra were obtained using the λ_{ex} specified in Table 1. From top to bottom and left to right, the spectra shown correspond to the probes ThT, ThS, pFTAA, hFTAA, ANS, Bis-ANS, X-34 and Congo Red, as specified by the titles on top of each. Light orange spectra represent the emission of the probes alone, while dark orange spectra indicate the emission obtained by the probe in the presence of TADC aggregates. The wavelengths corresponding to the main peaks are indicated. All spectra reflect the average of triplicates (line) with their standard deviation (shadow). See Materials and Methods for details.

Figure S2

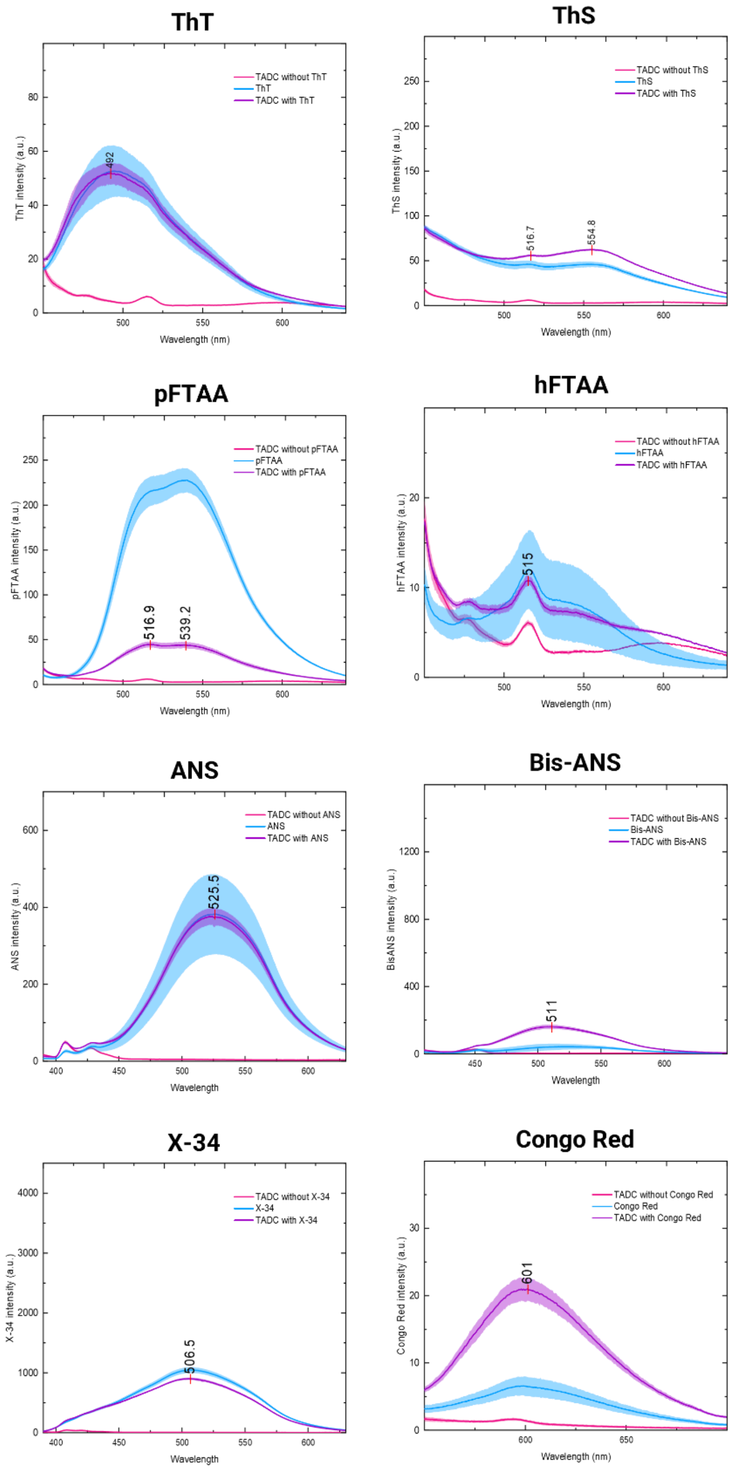

Figure S2. Emission spectra of fluorescent probes in the presence of TADC monomers. Each dye was added to 15 µM of TADC monomers in 20 mM sodium phosphate buffer pH 8.0, with 1 mM DTT and 1.1 mM $CaCl_2$. Spectra were obtained using the λ_{ex} specified in Table 1. Pink traces represent the emission spectra of TADC monomers alone; blue traces indicate the emission spectra of each individual probe; purple traces correspond to spectra of TADC monomers with each of the studied dyes. The emission maxima are indicated. Emission spectra were measured in triplicate (lines) with standard deviation represented as shadow. See Materials and Methods for details.
